# Marginal Bone Level and Clinical Parameter Analysis Comparing External Hexagon and Morse Taper Implants: A Systematic Review and Meta-Analysis

**DOI:** 10.3390/diagnostics13091587

**Published:** 2023-04-28

**Authors:** Samuele Fuda, Bruno Gomes dos Santos Martins, Filipe Correia de Castro, Artak Heboyan, Sergio Alexandre Gehrke, Juliana Campos Hasse Fernandes, Anna Carolina Volpi Mello-Moura, Gustavo Vicentis Oliveira Fernandes

**Affiliations:** 1 Faculty of Dental Medicine, Universidade Católica Portuguesa, 3504-505 Viseu, Portugal; 2 FP-I3ID, FCS, Universidade Fernando Pessoa, 4249-004 Porto, Portugal; 3 Department of Prosthodontics, Faculty of Stomatology, Yerevan State Medical University after Mkhitar Heratsi, Str. Koryun 2, Yerevan 0025, Armenia; 4 Department of Research, Bioface/PgO/UCAM, Calle Cuareim 1483, Montevideo 11100, Uruguay; 5 Instituto de Bioingenieria, Universidad Miguel Hernández, 03202 Elche, Spain; 6 Department of Biotechnology, Universidad Católica de Murcia (UCAM), 30107 Murcia, Spain; 7 Periodontics and Oral Medicine Department, University of Michigan School of Dentistry, Ann Arbor, MI 48109, USA; 8 Centre for Interdisciplinary Research in Health (CIIS), Universidade Católica Portuguesa, 3504-505 Viseu, Portugal

**Keywords:** clinical parameters, dental implants, dental implant–abutment connection, marginal bone loss, systematic review

## Abstract

The goal of this systematic review was to verify the marginal bone loss (MBL) and other clinical parameters comparing external hexagon (EH) and Morse taper (MT) implants when they were evaluated within the same study. The focused question was, “For patients (P) treated with external connection (I) or Morse taper (C) dental implants, were there differences in the marginal bone crest maintenance after at least three months in occlusal function (O)”? As for the inclusion criteria that were considered, they included clinical studies in English that compared the MBL in implants with EH and MT, with follow-up of at least three months, that were published between 2011 and 2022; as for the exclusion criteria, they included publications investigating only one type of connection that analyzed other variables and did not report results for the MBL, reports based on questionnaires, interviews, and case reports/series, systematic reviews, or studies involving patients with a significant health problem (ASA Physical Status 3 and above). The PubMed/MEDLINE, Embase, and Web of Science databases were screened, and all of the data obtained were registered in a spreadsheet (Excel^®^). The Jadad scale was used to assess the quality of the studies. A total of 110 articles were initially identified; 11 were considered for full-text reading. Then, six articles (four RCTs and two prospective studies) met the eligibility criteria and were included in this study. A total of 185 patients (mean age of 59.71) were observed, and the follow-up ranged from 3 months to 36 months. A total of 541 implants were registered (267 EH and 274 MT). The survival rate ranged between 96% and 100% (the average was 97.82%). The MBL was compared among all periods studied; therefore, the common assessment period was the 12-month follow-up, presenting greater MBL for EH than for MT (*p* < 0.001). A mean MBL of 0.60 mm (95% CI 0.43–0.78) was found after the same period. BoP was reported in 5 studies and plaque index was reported in 4 (2 with more than 30%). Deep PD was observed in three studies. High heterogeneity was observed (I_2_ = 85.06%). Thus, within the limitation of this review, it was possible to conclude that there is higher bone loss in EH than in MT implants when evaluating and comparing this variable within the same study. However, the results must be carefully interpreted because of this review’s limited number of clinical studies, the short assessment period, and the high heterogeneity found.

## 1. Introduction

Regarding the high success and survival rates found nowadays for dental implants, this treatment option has been considered to be a feasible tool to substitute lost teeth [1,2]. Modifications to the implants’ design and surface, considered to be two of the most important attributes, have been studied to enhance and accelerate the osseointegration process [3]. The changes in the implant’s topography and surface energy/tension can provide a proliferation-inducing and growth-originating surface. Moreover, the cells responsible for the tissue growth can enhance this phenomenon [4], with a direct, structural, and functional connection between the living bone tissue and the implant surface. This is the consequence of several molecular signaling pathways after implant placement [4], which is vital for long-term success and rehabilitation [5].

Nevertheless, several aspects can explain peri-implant bone loss. General factors involving the systemic condition include the patient’s age, overall health, socio-economic status, genetic factors, and oral hygiene habits, and local factors include occlusal overload, biological factors, implant design, and connection type [6]. Among them, biological factors (poor native bone quality, poor osseointegration due to biological reasons, or peri-implantitis) [7] are the most common causes of bone loss around implants. Peri-implantitis is a progressive biological disease leading to implant stability loss, deep pocket depth (PD), and the possible presence of suppuration [8,9]. Therefore, the current literature suggests that the connection type between implant and abutment (IA) plays a significant role in the long-term success of rehabilitation, since its influences the bony and soft tissue remodeling around the implant [10,11].

Biomechanically, the tension accumulated and dispersed in the connection can influence the marginal bone position, potentially leading to resorption/remodeling. Even though up to 1.5 mm of bone loss after the installation of a prosthetic component can be tolerated [12] within normal conditions, bone remodeling can negatively affect the gingival margin position and esthetic. However, this remodeling process typically ceases once the biological width is set [13,14]. Additionally, this “physiological” process can be an initial step in developing peri-implantitis since the IA interface has reduced protection and is more vulnerable to inflammatory cells [15,16]. In addition, a microscopical gap between IA (“void space”) can exist. This fact is a potential source of bacterial infection in this union [17], which may trigger the development of peri-implant diseases [18]. This fact is commonly observed in external hexagon (EH) implants, which have an IA interface located at the bone crestal level, affecting the surrounding bone stability and the quality of the gingival tissue [12,19].

Then, within this limitation of the EH system, manufacturers started to study other possibilities, mainly investing in platform switching. This type of connection is conical and is established inside the implant body. The implant’s diameter does not match the diameter of the abutment [17,20]. These features are found in the Morse taper implant system (MT). It is a microgap-free system, resulting in improved biomechanical behavior and a wider connective tissue attachment, protecting peri-implant tissues from microbial [21] and surface contamination and achieving better peri-implant bone maintenance [17]. Even after all improvements, studies revealed that conical internal connections could not avoid bacterial leakage [17,22].

However, despite the EH and MT implants having similar clinical indications, little information is available in the literature regarding the characteristics of the peri-implant tissue around the different prosthetic interfaces when assessed in the same study. Thus, considering the influence of the IA connection on marginal-bone-level changes, the goal of this systematic review was to verify, primarily, the marginal bone loss (MBL) between EH and MT, and secondarily, PD, bleeding on probing (BoP), and keratinized tissue width (KTW), comparing MT and EH.

## 2. Materials and Methods

### 2.1. Research Question

The literature considered for this systematic review was based on the PRISMA (Preferred Reporting Items for Systematic Review) guidelines [23] and we aimed to answer the following specific question constructed in the PICO (population, intervention, control, outcomes) format [24] (Table 1): “For patients (P) treated with external connection (I) or Morse taper (C) dental implants, were there differences in the marginal bone loss (MBL) after at least three months in occlusal function (O)”?

### 2.2. Inclusion and Exclusion Criteria

The inclusion criteria were (i) clinical studies that compared the marginal bone loss (MBL) in implants with external connection/hexagon (EH) and Morse taper (MT); (ii) follow-up of at least 3 months; and (iii) publications in the English language between January 2011 and 2022. The exclusion criteria were (i) publications investigating the types of desired connection individually; (ii) studies that analyzed other variables and did not report results for the MBL; (iii) reports based on questionnaires, interviews, and case reports/series, as well as systematic reviews; and (iv) studies involving patients with a significant health problem (ASA Physical Status 3 and above).

### 2.3. Study Search and Strategy of Selection

An electronic search was performed in the PubMed/MEDLINE, Embase, and Web of Science databases. The terms used in this search are detailed in Supplementary Table S1. A manual search was also performed on the articles obtained to find more articles that met the inclusion criteria. Two reviewers (SF and GVOF) independently assessed all the titles and abstracts retrieved from the electronic search to reach a consensus on excluding or admitting each study. In the case of disagreement, a third referee was consulted (JCHF). The Kappa value was reported to measure the inter-rater reliability for the qualitative items.

### 2.4. Data Extraction

A thorough analysis of the data was performed by two independent researchers (SF and GVOF) for sequential comparison in Microsoft^®^ Excel (v. 16.50, Microsoft Office, Redmond, WA, USA). The following information was extracted: information about the patients (age, gender, type and number of implants placed, number of patients treated); characteristics of the implants; surgical techniques adopted; whether the abutment was placed in one or in a second-stage procedure (two-stage/submerged) versus implants where the abutment was placed immediately (one-stage/non-submerged); implant loading (delayed versus immediate loading); type of prosthesis; implant placement time (socket healing versus immediate post-extraction); follow-up period; implant survival rate; and marginal bone loss (MBL). Regarding periodontal parameters, MBL, PD, BoP, KT, and radiographic data were registered when available. The implant survival rate was found when the implant was in place at the moment of the re-evaluation.

### 2.5. Quality Assessment and Statistical Analysis

Each study was assessed using the Jadad scale [25]. This assessment method consists of assessing the methodological quality of clinical trials. The score ranged between 0 and 5, where 0–2 were studies with low quality, 3–4 were studies with medium quality, and 5 was studies with high quality. The data were analyzed using a continuous random effects model meta-analysis. The studied quantitative variable was marginal bone loss. A forest plot was produced to graphically represent the difference in marginal bone loss results, comparing the control group (EH) and the test group (MT). The value of *p* = 0.05 was chosen to determine whether the differences were statistically significant. Heterogeneity was evaluated using the I_2_ test (R version 3.3.2, Foundation for Statistical Computing, Vienna, Austria; R studio version 1.0.44 Studio, Inc., Boston, MA, USA), whereby values between 0% and 40% were suggestive of no heterogeneity; values between 30% and 60% were suggestive of moderate heterogeneity; values between 50% and 90% were suggestive of substantial heterogeneity; and values between 75% and 100% were suggestive of high/considerable heterogeneity. The funnel plot was also developed to verify heterogeneity and whether studies were within the confidence interval (95%).

## 3. Results

### 3.1. Selection of Studies

**Figure 1:**
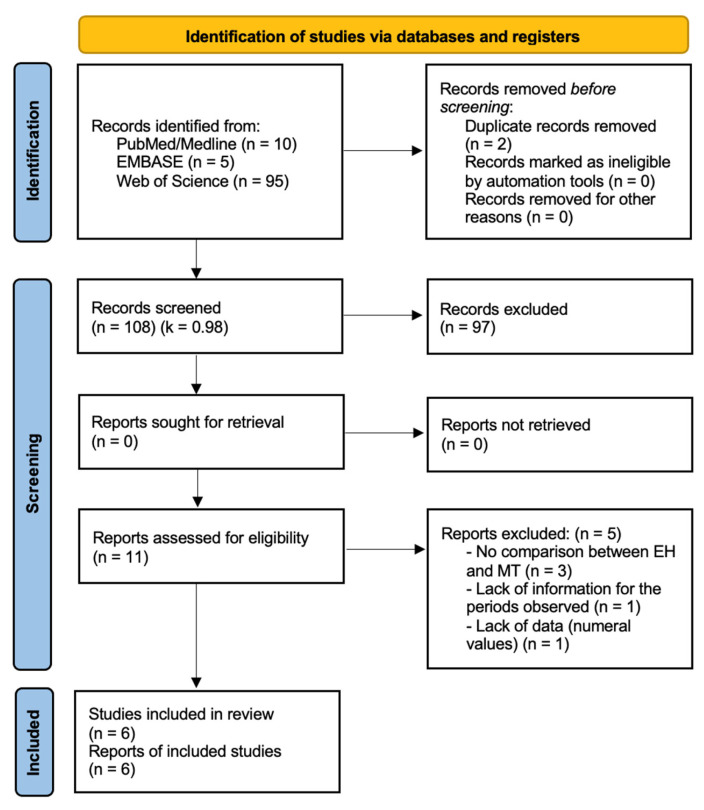
Flow chart of the screening and selection of the studies.

Initially, 110 articles were identified in electronic databases (5 in Embase; 10 in PubMed/MedLine; and 95 in Web of Science). Of the 110 articles found through the search strategy, 2 duplicate articles were removed, and the remaining 108 were reviewed according to title and abstract. Upon review of the title and abstract, 95 articles that did not meet the inclusion criteria were excluded. The remaining 11 articles were considered for full-text reading, which led to the exclusion of another 5 articles according to the application of the inclusion and exclusion criteria (3 articles were excluded due to the lack of comparison between the types of connections studied in this review; 1 was excluded because of the lack of information according to the periods observed; and another 1 was excluded due to a lack of numerical data provided). The remaining six articles met the inclusion and exclusion criteria. They were included in the study (Figure 1). The designs presented were four randomized and controlled clinical trials (RCTs) [26,27,28,29] and two prospective studies (PSs) [30,31]. A total of 3 out of 6 studies (50%) had a split-mouth design, 4 were RCTs (66.66%), and 2 were PSs with 1 year [30] and 3 years [30] of follow-up.

### 3.2. Characteristics of the Patients Observed (Table 2)

A total of 185 patients with a mean age of 59.71 years old (between 18 and 83 years old) were observed, and the follow-up period ranged from 3 months to 36 months. Only 1 study did not mention the gender of the patients [26], while 111 patients were correctly sorted (64 men and 47 women). All the included articles [26,27,28,29,30,31] reported the exclusion/drop-off of patients and gave reasons. Twenty patients were excluded due to unfavorable anatomical conditions or pragmatic factors, six patients were unavailable for follow-up, three spontaneously discontinued the study, and one did not show up for prosthesis delivery.

### 3.3. Characteristics of the Implants and Survival Rate (Table 3)

A total of 541 implants were placed; however, 1 study (42) did not report the placement site. Otherwise, 403 implants were identified (217 in the maxilla and 186 in the mandible). Of the 541 implants (153 participants), 267 had EH design and 274 had internal conical/Morse taper connections. The survival rate ranged between 96% and 100% (an average of 97.82%). Only 1 study [31] reported the success rate, considering implants with MBL lower than 2 mm. All the studies, excluding Copper et al.’s [30], which did not report details about the implant dimensions, used regular platform implants, with varying lengths between 9 mm and 13 mm.

### 3.4. Clinical Findings (Table 4 and Table 5)

The MBL was compared among all periods (3, 6, 12, 21, and 26 months). For 3 months, only Pozzi et al. [26] and Glibert et al. [29] reported data with higher MBL in the EH group; otherwise, there were reduced values when the EH implant presented microthreads [29]. After 6 months, 3 studies were compared: Glibert et al. [29] and Doornewaard et al. [27] had similar results among the groups, whereas Penarrocha-Diago et al. [31] found greater MBL in the EH group. At the 12-month follow-up, the only finding common to all studies was that the EH had greater MBL than the MT group (Table 4). Cooper et al. [30] and Doornewaard et al. [27] showed the most prolonged period, which also showed increased MBL for the EH group.

BoP was found in five out of the six studies included. A non-significant result (no bleeding or <10%) was found by Cooper et al. [30], Pozzi et al. [26], and Pessoa et al. [28]. However, Doornewaard et al. [27] and Glibert et al. [29] presented moderate bleeding levels, respectively, in 41 implants (41.84%) and 24.4% of cases. The quantity of plaque was observed in 4 studies; 2 had more than 30% registered. The PD variable around implants (EH and Morse) was observed in three studies: Doornewaard et al. [27], Glibert et al. [29], and Pessoa et al. [28]. The mean findings were 4.5 mm/2.1 mm, 3.26 mm, and 1.57 mm/1.36 mm, respectively.

### 3.5. Quality Assessment and Statistical Analysis

**Figure 2:**
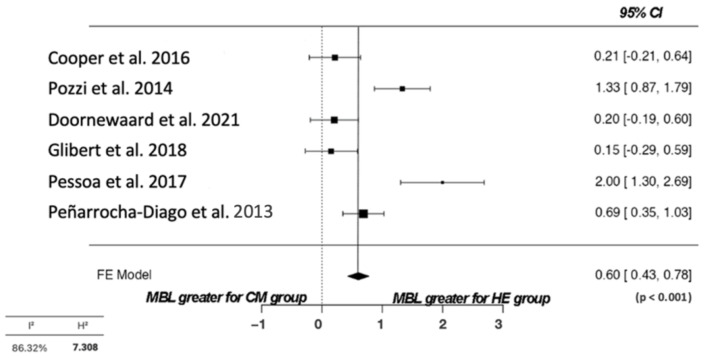
Forest plot comparing the groups under analysis for marginal bone loss for 12 months [26,27,28,29,30,31].

**Figure 3:**
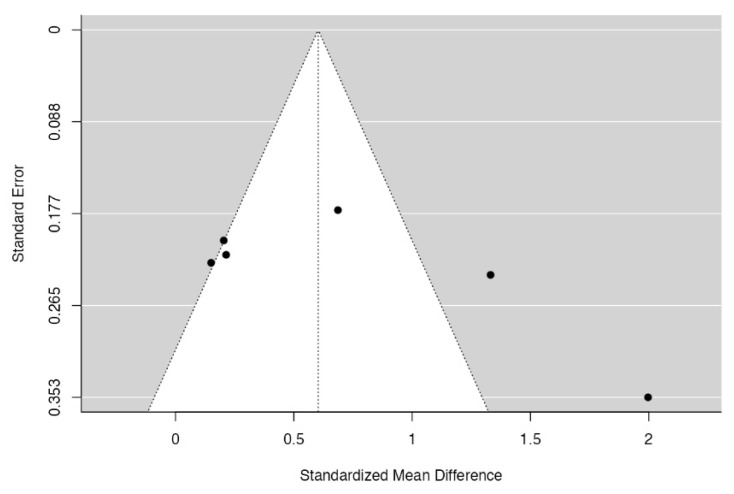
A funnel plot was performed to analyze the study groups for 12 months.

The quality assessment showed that all investigations had an overall medium quality. Only the study by Pozzi et al. [26] had high quality, with a double-blind design, and correctly reported blind adequacy (Table 6). The meta-analysis (only for 12 months) showed via a forest plot (Figure 2) that there was a statistically significant result for the MBL between groups (*p* < 0.001). The most significant loss was registered in the EH implant group. Furthermore, there was high heterogeneity between studies (I_2_ = 85.06%), which could be confirmed via a funnel plot (Figure 3). A mean peri-implant bone loss of 0.60 mm (95% CI 0.43–0.78) was found at the 1-year follow-up.

## 4. Discussion

Even though the implant survival rate may have achieved more than 90% in the long-term [2,32,33], the presence of MBL may permit implant thread exposition, leading to an easy bacterial accumulation and consequent peri-implant diseases, with possible implant loss [8,34,35]. The objective of this systematic review was to answer a specific clinical question, comparing the results between EH and MT implants when assessed within the same study (reducing bias). This topic is crucial, focusing on the longevity of dental implants and the clinical predictability of the tissue around them, mainly crestal bone maintenance. Hence, bone loss related to the 2 types of implant connections and other clinical parameters were analyzed, considering a follow-up period between 3 months and 3 years.

Albrektsson et al. [36] proposed specific criteria to standardize crestal bone remodeling. They considered acceptable, marginal bone remodeling to be up to −2.0 mm in the 1st year after loading and up to −0.2 mm each year thereafter. These changes are usually related to implants placed at the bone level, with a conventional machined surface and conventional neck design. In addition, the remodeling was assessed regardless of the surgical procedure adopted, the position of the implant, or its characteristics [36]. Thus, even with a well-positioned implant, there is a trend predicted to happen of MBL of 2 mm during the 1st year and approximately 0.2 mm for each subsequent year, which is considered to be acceptable [37,38]. Therefore, the resorption of marginal bone does not begin until the implant is uncovered and exposed to the oral cavity, which permits bacterial contamination [39,40,41,42,43].

In the present systematic review, MT and EH connections showed bone remodeling during the first year of loading. However, significantly higher MBL values were found in EH implants. The average MBL was −0.62 mm for EH connections and −0.26 mm for MT implants after 1 year, which was lower than that reported in the literature. Likewise, several clinical studies in the literature have evaluated the influence of the type of connection on MBL, suggesting similar observations for EH implants compared to internal connections [44,45]. Our findings agree with Camps-Font et al. [44], who showed less MBL after one-year follow-up, and Kim et al. [45], who found lower MBL for MT than EH. Oppositely, two RCTs [46,47] did not find clinical differences for MBL between that which was platform-switched and that which was non-platform-switched.

Furthermore, MBL around MT implants had less bacterial infiltration, and, consequently, less local unbalance. The microgap is a critical parameter that must be better evaluated, even though this was not the aim in this systematic study. It may enhance bone resorption due to mechanical micromovements or bacterial contamination. This contamination inside the microgap depends on the fit of the MT or EH at the implant–abutment interface, which may result in bacterial flow and initiate an inflammatory response around the implant [48]. This phenomenon [42] was a biological protective mechanism against the bacteria in that site. It can explain the plaque-dependent MBL, around 1 to 2 mm, observed during the 1st year. Then, placing the microgap away from the implant shoulder is suggested, different from that observed in the EH, increasing its distance from the bone [49,50,51]. This method generally implies using a reduced-diameter abutment, such as in the MT connection, to protect the marginal bone.

Besides the points mentioned above, the bone remodeling will continue until the “recreation” and stabilization of the supracrestal tissue attachment (“biologic width”), which achieves an average between 1 and 2 mm of circumferential bone remodeling in the 1st year of function [52]. Furthermore, it is necessary to respect the distance between the bony base of the papilla and the contact point of the teeth/crowns, which should be up to 5 mm, to expect the complete filling of the interdental space with gingival tissue to form normal papillae [53,54]. Understanding the differences between peri-implantitis and normal tissue remodeling is essential. The first is a progressive biological disease, causing bone loss, increasing the PD, and possibly presenting suppuration [8,9]. Many surgical and non-surgical treatments for peri-implant mucositis and peri-implantitis have been proposed and evaluated [8,55], with scientific results supporting better responses to surgical procedures [8].

New strategies have been developed to assess the improvement in the osseointegration and tissue adaptation to the biomaterials used [56]. The literature also suggests that the treatment or modification of the implant surface can ensure less MBL, regardless of the type of connection, reducing the peak stress due to the rough surface [57,58,59]. This could explain the elevated MBL related to external connections, as observed in Penarocha-Diago et al.’s [31] study, which had different implant surfaces (machined) in the EH and microthreads in the internal connection. In addition, two more articles [27,29] included in this review also evaluated the MBL in implants with different surface compositions (with and without microthreads). Both reported non-significant MBL between the connections and different implant microdesigns. The possible justification may be due to a relatively short-term follow-up applied to assess the crestal bone maintenance. In a long-term prospective study [58] at 1, 3, and 5 years of follow-up, the MBL was relatively lower around implants with microthreads. This fact was corroborated by Al-Thobity et al. [60], in a systematic study, who reported that microthreaded dental implants were a better choice than implants with other designs.

### 4.1. Other Clinical Parameters

Another factor that must be well observed and is hard to be controlled in systematic studies (a confounding variable) is the surgical trauma caused by the implant placement procedure. This factor can cause MBL [61]. In particular, the excessive torque force used can cause vascular impairment and peri-implant bone remodeling, contributing to necrosis and causing microfractures in the cortical bone [62]. In the studies by Glibert et al. [29] and Doorneward et al. [27], the insertion forces of 20 N.cm were respected, and no differences were found between the treated groups. A prospective study [63] showed that, using torque force < 20 N.cm, there were higher success rates and stability during osseointegration and reduced damage to the surrounding tissues, resulting in reduced changes in the marginal bone in the short- and mid-term.

Other clinical parameters evaluated in this study were BoP, PD, and plaque index. There was a limitation in assessing these variables, impairing the direct comparison of values according to the type of connection studied. Indeed, some studies have reported general results without distinguishing them by the type of connection. In the included studies, the results for these parameters did not reveal significant differences between EH and MT within the periods analyzed. From this perspective, our clinical results agree with Esposito et al. [64], who assessed internal conical and EH, presenting no statistically significant differences in clinical outcomes. Moreover, the authors suggested that the type of connection may simply be based on the physician’s preference.

On the other hand, a comparative study [65] found significant differences in clinical parameters at 3 months and 1 year. The MT implants showed significantly reduced peri-implant inflammation, which was not observed in the EH group. Additionally, it was highlighted that biofilm accumulation on the abutments of the MT group could be explained due to the abutment position below the alveolar bone crest. Moreover, comparing the MBL between fixed (found in this study) and removable (overdenture) rehabilitation, the values were greater in the removable rehabilitation, with the stud-retentor presenting the greatest MBL (−1.96 mm) and the bar-clip system having mean MBL of −1.13 mm [66].

### 4.2. Study Limitations

The present systematic article has some limitations: (i) a low number of clinical studies were included, which compared EH and Morse taper implants in the same study (RCT or controlled clinical trial); (ii) only 1 article met the high-quality assessment; (iii) the position of the implant regarding the marginal level was different between the EH (bone level) and Morse taper (infrabony), which became difficult for the appraisal, even though the initial and final radiographic evaluations kept the same standard baseline; and (iv) the statistical data were limited to 12-month follow-up. Moreover, we recommend future simulation in medical investigations using implants, similar to a recent publication [67], offering advantages such as lower cost and faster results compared to clinical studies.

## 5. Conclusions

Despite the present limitations, it is possible to conclude there is higher bone maintenance needed for MT rather than for EH implants at 12 months after implant placement. However, the results must be carefully interpreted due to the limited number of clinical studies included in this review, with a short assessment period and high heterogeneity. In addition, new well-standardized investigations are needed, including a larger number of patients/implants, analyzing EH and MT in the same study, and having a longer follow-up period, such as 3, 5, and 10 years, to better compare the alveolar bone crest and adequate control of confounding variables.

## Figures and Tables

**Table 1:** PICO question.

Population	Patients Treated with Dental Implants
**Intervention**	Implants with external connection
**Control**	Implants with Morse taper
**Outcomes**	Differences in the marginal crestal bone (maintenance) after at least three months of function

**Table 2:** Objectives of the included studies and patient details.

Author/Year	Study Design	Objective	*n*	Age	Gender	Smokers
				Mean/Range	Male/Female	
Pozzi et al., 2014 [26]	RCT (split-mouth)	Compare clinical and radiological outcomes of two implant designs with different prosthetic interfaces and neck configurations in a randomized, controlled, split-mouth clinical trial.	34	52.20/39–59	NR	4 patients smoke less than 10 cigarettes/day
Doornewaard et al., 2021 [27]	RCT (split-mouth)	They assessed the effect of implant neck (microthreaded vs. non-microthreaded) and the type of abutment connection (internal conical vs. external flat-to-flat) on peri-implant bone stability and peri-implant health after at least 36 months.	27	62/42–83	M:15 F:12	Limited to patients smoking less than 10 cigarettes/day
Pessoa et al., 2017 [28]	RCT (split-mouth)	To evaluate clinical, radiographic, microbiologic, and biomechanical parameters related to bone remodeling around implants with external hexagon (EH) and Morse taper (MT) connections.	12	63/18–75	M:3 F:9	0
Glibert et al., 2018 [29]	RCT	This RCT assesses whether a coronal microthreaded design and an internal abutment connection affect crestal bone loss up to one year of function.	21	65/44–66	M:12 F:9	Limited to patients smoking less than 10 cigarettes/day
Cooper et al., 2016 [30]	PS	Over 3 years, compare the proximal marginal bone responses at external hex interface (EXI) versus internal conus interface (ICI) implants.	36	53.1/18–75	M:13 F:23	14 previous smokers
Peñarrocha-Diago et al., 2013 [31]	PS	To conduct a comparative study of two implants with different neck features and prostheses platform connection (machined with external connection and rough-surfaced with switching platform) upon peri-implant marginal bone loss, before and after functional loading.	15	56.9/44–77	M:4 F:11	3 smoking patients, less than 10 cigarettes/day

PS: prospective study; RCT: randomized controlled trial; vs.: versus; NR: not reported.

**Table 3:** Information on the surgical site, implants, procedures, and follow-up.

Author/Year	Surgical Site	Implant Loading	Implant/Abutment Loading	Follow-Up (Months)	Implants								
					*n*	Location	Groups Treated	Threads	Length (mm)	Width (mm)	Manufacturer	Success Rate (%)	Survival Rate (%)
Pozzi et al., 2014 [26]	Any partially edentate patient in the lower jaw, aged 25 years or more, requiring at least two single implant-supported crowns; sufficient bone volumes to accommodate dental implants without augmentation procedure.	D	2nd stage	8 weeks; 4; 16	88	Max: 0 Mand: 88	Test: ICC Control: EH	MT MT	10–13 10–13	4.3 4	Nobel Active, Nobel Biocare AB Nobel Speedy Groovy, Nobel Biocare AB	NR	100
Doornewaard et al., 2021 [27]	Fully edentulous patients in the maxilla in need of a four-implant-supported overdenture. The preferred implant locations were the canine and first-molar regions. In case of insufficient bone in the molar region, the second premolar site was chosen.	I	1st stage	3; 6; 12; 24; 36	98	Max: 98 Mand: 0	I-MT I-NMT E-MT I-NMT	MT NMT MT NMT	9–11 9–11 9–11 9–11	4 4 4 4	DCC, Southern implants Southern implants	98.4	95.9
Pessoa et al., 2017 [28]	Edentulous patients should also have adequate bone quantity for the placement of 4 3.8 and 3 13 mm implants in the interforaminal region of the mandible.	I	1st stage	1; 3; 6; 12	48	Max: 0 Mand: 48	Test: Morse taper connection Control: External hexagon connection	MT MT	NR NR	NR NR	UNITITE^®^, SIN	NR	100
Glibert et al., 2018 [29]	Totally edentulous patients in the upper jaw for at least 4 months; the presence of sufficient residual bone volume to install 4 implants with a 4 mm diameter and 9–11 mm in length.	I	1st stage	3; 6; 12; 21	83	Max: 83 Mand: 0	I-MT I-NMT E-MT I-NMT	MT NMT MT NMT	9–11 9–11 9–11 9–11	4 4 4 4	SICace^®^; SIC invent Southern implants	NR	96.4
Cooper et al., 2016 [30]	Individuals classified as Kennedy class I or II for mandibular or maxillary arches involving the left, the right, or both quadrants were eligible for enrollment.	D	1st stage	6; 12; 36	86	Max: 36 Mand: 50	Test: ICI Control: EXI	NR	NR	NR	Osseotite Standard, Biomet 3i Astra Tech Fixture ST, Dentsply	NR	96
Penarrocha-Diago et al., 2013 [31]	Completely edentulous arch requiring implant placement for a fixed prosthesis, bar overdenture, locator overdenture; bone with a minimum width of 7 mm and a minimum height of 6 mm.	D	2nd stage	Implant placement and prosthesis placement: 6; 12	141	NR	Group A: External hexagon Group B: Internal connection and platform switching	NMT MT	10–13 10–13	3.75–4.25 3.75–4.25	Osseous^®^, Mozo-Grau Inhex^®^, Mozo-Grau	97.2	98.6

D: delayed; I: immediate; NR: not reported; Max: maxilla; Mand: mandible; ICI: internal conus design implants; ICC: internal conical connection with back-tapered collar and platform shifting; EXI: external hexagon design implants; EH: external hexagon with a flat-to-flat interface; MT: microthreads; I-MT: internal with microthreads; I-NMT: internal without microthreads; E-MT: external with microthreads; E-NMT: external without microthreads.

**Table 4:** Marginal bone loss (MBL) assessment.

Author/Year	Patients (*n*)	Implants (*n*)	Morse Taper				
			3 m	6 m	12 m	21 m	36 m
Pozzi et al., 2014 [26]	34	88	−0.37 ± 0.23	NR	−0.51 ± 0.34	NR	NR
Doornewaard et al., 2021 [27]	27	98	NR	I-MT −0.45 ± 0.61 t0–t1	I-MT −0.01 ± 0.47, t1–t2	NR	I-MT −0.01 ± 0.47, t1–t2
				I-NMT −0.33 ± 0.61 t0–t1	I-NMT −0.07 ± 0.60, t1–t2		I-NMT −0.07 ± 0.60, t1–t2
Pessoa et al., 2017 [28]	12	48	NR	NR	−0.17 ± 0.54	NR	NR
Glibert et al., 2018 [29]	21	83	I-MT −0.27 ± 0.65	I-MT −0.34 ± 0.47	I-MT −0.22 ± 0.32	I-MT −0.26 ± 0.32	NR
			I-NMT −0.15 ± 0.29	I-NMT −0.26 ± 0.39	I-NMT −0.27 ± 0.42	I-NMT −0.24 ± 0.36	
Cooper et al., 2016 [30]	36	86	NR	NR	−0.48 ± 0.55	NR	−0.25 ± 0.60
Penarrocha-Diago et al., 2013 [31]	15	141	NR	−0.07 ± 0.13 mm	−0.12 ± 0.17 mm	NR	NR
**External Hexagon**							
**3 m**	**6 m**		**12 m**		**21 m**	**36 m**	
−0.95 ± 0.56	NR		−1.10 ± 0.52		NR	NR	
NR	E-MT −0.45 ± 0.77, t0–t1		E-MT −0.10 ± 0.58 t1–t2		NR	E-MT −0.10 ± 0.58, t1–t2	
	E-NMT −0.34 ± 0.51, t0–t1		E-NMT −0.19 ± 0.48, t1–t2			E-NMT −0.19 ± 0.48, t1–t2	
NR	NR		−1.17 ± 0.44		NR	NR	
E-MT −0.24 ± 0.36	E-MT −0.32 ± 0.42		E-MT −0.32 ± 0.45		E-MT −0.22 ± 0.33	NR	
E-NMT −0.16 ± 0.25	E-NMT −0.29 ± 0.36		E-NMT −0.29 ± 0.38		E-NMT −0.19 ± 0.23		
NR	NR		−0.68 ± 1.2		NR	−0.5 ± 0.93	
NR	−0.27 ± 0.43		−0.38 ± 0.51		NR	NR	

I-MT—internal with microthreads, I-NMT—internal without microthreads, E-MT—external with microthreads, E-NMT—external without microthreads, NR—not reported.

**Table 5:** Clinical parameters found.

Author/Year	Patients (*n*)	Connection (*n*)	BoP	PD (mm)	Plaque	Follow-Up (Months)	Conclusions
Pozzi et al., 2014 [26]	34	ICC (44)	Not detected around any implants	NR	Low presence	16	The MBL was statistically significantly lower in the back-tapered neck configuration with CC and built-in platform shifting compared with the straight neck configuration with the flat-to-flat implant–abutment interface and external hexagonal connection.
		EH (44)	Not detected around any implants		Low presence		
Doornewaard et al., 2021 [27]	27	I-MT (24)	Positive in 33 implants	Mean of 4.5	No significant impact between implant type and position	36	The implant–abutment connection (internal vs. external), implant neck design (microthreaded vs. non-microthreaded), and implant position (anterior vs. posterior) have no influence on peri-implant bone remodeling after implant placement, no impact on peri-implant bone level after initial remodeling, and no effect on peri-implant health parameters.
		I-NMT (25)					
		E-MT (25)					
		E-NMT (24)					
		Platform matching	Positive in 8 sites	Mean of 2.1	36 implants with plaque		
Pessoa et al., 2017 [28]	12	External hexagon (24)	No bleeding	1.57 ± 0.9	NR	12	Within the limitations of this study, it can be concluded that varying implant–abutment connection types will result in diverse early peri-implant bone remodeling. The present findings suggest that MT connections are more efficient in preventing early peri-implant bone loss compared to EH connections.
		Morse Taper (24)	No bleeding	1.36 ± 0.7			
Glibert et al., 2018 [29]	21	I-MT (20)	23.4% was recorded	Mean of 3.26	39.5% of implants presented the plaque	12–21	From this RCT, it is concluded that crestal bone remodeling is not affected by the implant–abutment connection or microthreads. Bone remodeling is a multifactorial process and might be more dependent on other factors than implant design itself.
		I-NMT (21)					
		E-MT (20)					
		E-NMT (19)					
Cooper et al., 2016 [30]	36	ICI (44)	Less than 2%	NR	Low presence	36	Comparing two implant designs revealed minor differences in marginal bone responses from permanent restoration to 3 years. Significantly more apical MBLs were recorded for EXI implants. Furthermore, more positive papilla scores were found between adjacent ICI implants than between adjacent EXI implants. EXI implant displayed more abutment complications than the ICI implant.
		EXI (42)	Less than 2%		Low presence		
Penarrocha-Diago et al., 2013 [31]	15	EH (69)	NR	NR	NR	12	Bone loss after 6 and 12 months proved statistically significant between the two groups, with comparatively greater loss in the case of the Osseous^®^ implants vs. the Inhex^®^ implants. Regardless of the heterogeneity of the two groups (neck shape, microthreads, surface texture), the implant–abutment connection appears to be a significant factor in peri-implant crestal bone levels.
		IC (72)					

vs.: versus; NR: non-reported; RCT: randomized and controlled trial; ICI: internal conus design implants; ICC: internal conical connection with back-tapered collar and platform shifting; EXI: external hexagon design implants; EH: external hexagon with the flat-to-flat interface; MT: microthreads; I-MT: internal with microthreads; I-NMT: internal without microthreads; E-MT: external with microthreads; E-NMT: external without microthreads.

**Table 6:** Quality assessment using Jadad scale.

Author/Year	Randomization	Appropriateness of Randomization	Blinding	Appropriateness of Blinding	An Account of All Patients or Description of Withdrawal or Drop-Out	Total
Pozzi et al., 2014 [26]	1	1	1	1	1	5
Doornewaard et al., 2021 [27]	1	1	0	0	1	3
Pessoa et al., 2017 [28]	1	0	1	1	1	4
Glibert et al., 2018 [29]	1	1	0	0	1	3
Cooper et al., 2016 [30]	1	1	0	0	1	3
Peñarrocha-Diago et al., 2013 [31]	1	1	0	0	1	3

## Supplementary Materials

The following supporting information can be downloaded at https://www.mdpi.com/article/10.3390/diagnostics13091587/s1: Table S1: Search strategy carried out and filters applied.

## References

[1] Alghamdi H.S., Jansen J.A. (2020). The development and future of dental implants. Dent. Mater. J..

[2] Borges H., Correia A.R.M., Castilho R.M., Fernandes G.V.O. (2020). Zirconia Implants and Marginal Bone Loss: A Systematic Review and Meta-Analysis of Clinical Studies. Int. J. Oral. Maxillofac. Implant..

[3] Gehrke S.A., Cortellari G.C., Fernandes G.V.O., Scarano A., Martins R.G., Cançado R.M., Mesquita A.M.M. (2023). Randomized Clinical Trial Comparing Insertion Torque and Implant Stability of Two Different Implant Macrogeometries in the Initial Periods of Osseointegration. Medicina.

[4] Pellegrini G., Francetti L., Barbaro B., del Fabbro M. (2018). Novel surfaces and osseointegration in implant dentistry. J. Investig. Clin. Dent..

[5] Esposito M., Hirsch J.M., Lekholm U., Thomsen P. (1998). Biological factors contributing. Eur. J. Oral Sci..

[6] Naveau A., Shinmyouzu K., Moore C., Avivi-Arber L., Jokerst J., Koka S. (2019). Etiology and measurement of peri-implant crestal bone loss (CBL). J. Clin. Med..

[7] Kowalski J., Lapinska B., Nissan J., Lukomska-Szymanska M. (2021). Factors influencing marginal bone loss around dental implants: A narrative review. Coatings.

[8] Martins B.G.S., Fernandes J.C.H., Martins A.G., Castilho R.M., Fernandes G.V.O. (2022). Surgical and Nonsurgical Treatment Protocols for Peri-implantitis: An Overview of Systematic Reviews. Int. J. Oral. Maxillofac. Implant..

[9] Smeets R., Henningsen A., Jung O., Heiland M., Hammächer C., Stein J.M. (2014). Definition, etiology, prevention and treatment of peri-implantitis—A review. Head Face Med..

[10] Caricasulo R., Malchiodi L., Ghensi P., Fantozzi G., Cucchi A. (2018). The influence of implant-abutment connection to peri-implant bone loss: A systematic review and meta-analysis. Clin. Implant. Dent. Relat. Res..

[11] Carossa M., Alovisi M., Crupi A., Ambrogio G., Pera F. (2022). Full-Arch Rehabilitation Using Trans-Mucosal Tissue-Level Implants with and without Implant-Abutment Units: A Case Report. Dent. J..

[12] Arvidson K., Fartash B., Hilliges M., Kondell P.A. (1996). Histological characteristics of peri-implant mucosa around branemark and 429 single-crystal sapphire implants. Clin. Oral Implant. Res..

[13] Qian J., Wennerberg A., Albrektsson T. (2012). Reasons for Marginal Bone Loss around Oral Implants. Clin. Implant. Dent. Relat. Res..

[14] Bio-Joachim SHermann C.D., Higginbottom F.L., Cochran D.L. (2000). Biologic width around titanium implants. A physiologically formed and stable dimension over time. Clin. Oral Implant. Res..

[15] Vigolo P., Gracis S., Carboncini F., Mutinelli S. (2016). Internal- vs. External-Connection Single Implants: A Retrospective Study in an Italian Population Treated by Certified Prosthodontists. Int. J. Oral Maxillofac. Implant..

[16] Lemos C.A.A., Verri F.R., Bonfante E.A., Santiago Júnior J.F., Pellizzer E.P. (2018). Comparison of external and internal implant-abutment connections for implant supported prostheses. A systematic review and meta-analysis. J. Dent..

[17] Sasada Y., Cochran D. (2017). Implant-Abutment Connections: A Review of Biologic Consequences and Peri-implantitis Implications. Int. J. Oral Maxillofac. Implant..

[18] Canullo L., Penarrocha-Oltra D., Soldini C., Mazzocco F., Penarrocha M., Covani U. (2015). Microbiological assessment of the implant-abutment interface in different connections: Cross-sectional study after 5 years of functional loading. Clin. Oral Implant. Res..

[19] Adell R., Lekholm U., Rockler B., Branemark P.-I., Lindhe J., Eriksson B., Sbordone L. (1986). Marginal tissue reactions at osseoin- 427 tegrated titanium fixtures: A 3-year longitudinal prospective study. Int. J. Oral Maxillofac. Surg..

[20] Gupta S., Sabharwal R., Nazeer J., Taneja L., Choudhury B., Sahu S. (2019). Platform switching technique and crestal bone loss around the dental implants: A systematic review. Ann. Afr. Med..

[21] Berglundh T., Lindhe J., Jonsson K., Ericsson I. (1994). The topography of the vascular systems in the periodontal and peri-implant 433 tissues in the dog. J. Clin. Periodontol..

[22] Filho A.P.R., Fernandes F.S.F., Straioto F.G., Silva W.J., Del Bel Cury A.A. (2010). Preload Loss and Bacterial Penetration on Different Implant-Abutment Connection Systems. Braz. Dent. J..

[23] Page M.J., McKenzie J.E., Bossuyt P.M., Boutron I., Hoffmann T.C., Mulrow C.D., Shamseer L., Tetzlaff J.M., Akl E.A., Brennan S.E. (2021). The PRISMA 2020 statement: An updated guideline for reporting systematic reviews. BMJ.

[24] Miller S.A., Forrest J.L. (2001). Enhancing your practice through evidence-based decision making: PICO, learning how to ask good questions. J. Evid. Based Dent. Pract..

[25] Jadad A.R., Andrew Moore R., Carroll D., Jenkinson C., John Reynolds D.M., Gavaghan D.J., McQuay H.J. (1996). Assessing the Quality of Reports of Randomized Clinical Trials: Is Blinding Necessary?. Control. Clin. Trials.

[26] Pozzi A., Agliardi E., Tallarico M., Barlattani A. (2014). Clinical and radiological outcomes of two implants with different prosthetic interfaces and neck configurations: Randomized, controlled, split-mouth clinical trial. Clin. Implant. Dent. Relat. Res..

[27] Doornewaard R., Sakani S., Matthys C., Glibert M., Bronkhorst E., Vandeweghe S., Vervaeke S., De Bruyn H. (2021). Four-implant-supported overdenture treatment in the maxilla. Part I: A randomized controlled split mouth trial assessing the effect of microthreads and abutment connection type on 4 years peri-implant health. Clin. Implant. Dent. Relat. Res..

[28] Pessoa R.S., Sousa R.M., Pereira L.M., Neves F.D., Bezerra F.J.B., Jaecques S.V.N., Sloten J.V., Quirynen M., Teughels W., Spin-Neto R. (2017). Bone Remodeling Around Implants with External Hexagon and Morse-Taper Connections: A Randomized, Controlled, Split-Mouth, Clinical Trial. Clin. Implant. Dent. Relat. Res..

[29] Glibert M., Vervaeke S., Jacquet W., Vermeersch K., Östman P.O., de Bruyn H. (2018). A randomized controlled clinical trial to assess crestal bone remodeling of four different implant designs. Clin. Implant. Dent. Relat. Res..

[30] Cooper L., Tarnow D., Froum S., Moriarty J., de Kok I. (2016). Comparison of Marginal Bone Changes with Internal Conus and External Hexagon Design Implant Systems: A Prospective, Randomized Study. Int. J. Periodontics Restor. Dent..

[31] Peñarrocha-Diago M.A., Flichy-Fernández A.J., Alonso-González R., Peñarrocha-Oltra D., Balaguer-Martínez J., Peñarrocha-Diago M. (2013). Influence of implant neck design and implant-abutment connection type on peri-implant health. Radiological study. Clin. Oral Implant. Res..

[32] Fernandes G.V.O., Costa B.M.G.N., Trindade H.F., Castilho R.M., Fernandes J.C.H. (2022). Comparative analysis between extra-short implants (≤6 mm) and 6 mm-longer implants: A meta-analysis of randomized controlled trial. Aust. Dent. J..

[33] Fernandes P.R.E., Otero A.I.P., Fernandes J.C.H., Nassani L.M., Castilho R.M., Fernandes G.V.O. (2022). Clinical Performance Comparing Titanium and Titanium–Zirconium or Zirconia Dental Implants: A Systematic Review of Randomized Controlled Trials. Dent. J..

[34] Fernandes G.V.O. (2021). Peri-implantitis matter: Possibilities of treatment but without a strong predictability for solution. Environ. Dent. J..

[35] Srimaneepong V., Heboyan A., Zafar M.S., Khurshid Z., Marya A., Fernandes G.V.O., Rokaya D. (2022). Fixed Prosthetic Restorations and Periodontal Health: A Narrative Review. J. Funct. Biomater..

[36] Albrektsson T., Zarb G., Worthington P., Eriksson R.A. (1986). The long-term efficacy of currently used dental implants: A review and proposed criteria for success. Int. J. Oral Maxillofac. Implant..

[37] Lesaffre E., Philstrom B., Needleman I., Worthington H. (2009). The design and analysis of split-mouth studies: What statisticians and clinicians should know. Stat. Med..

[38] Palaska I., Tsaousoglou P., Vouros I., Konstantinidis A., Menexes G. (2016). Influence of placement depth and abutment connection pattern on bone remodeling around 1-stage implants: A prospective randomized controlled clinical trial. Clin. Oral Implant. Res..

[39] Tarnow D.P., Cho S.C., Wallace S.S. (2000). The effect of inter-implant distance on the height of inter-implant bone crest. J. Periodontol..

[40] Quirynen M., van Steenberghe D. (1993). Bacterial colonization of the internal part of two-stage implants. An in vivo study. Clin. Oral Implant. Res..

[41] Quirynen M., Bollen C.M., Eyssen H., van Steenberghe D. (1994). Microbial penetration along the implant components of the Brånemark system. An in vitro study. Clin. Oral Implant. Res..

[42] Ericsson I., Persson L.G., Berglundh T., Marinello C.P., Lindhe J., Klinge B. (1995). Different types of inflammatory reactions in peri-implant soft tissues. J. Clin. Periodontol..

[43] Persson L.G., Lekholm U., Leonhardt A., Dahlén G., Lindhe J. (1996). Bacterial colonization on internal surfaces of Brånemark system implant components. Clin. Oral Implant. Res..

[44] Camps-Font O., Rubianes-Porta L., Valmaseda-Castellón E., Jung R.E., Gay-Escoda C., Figueiredo R. (2021). Comparison of external, internal flat-to-flat, and conical implant abutment connections for implant-supported prostheses: A systematic review and network meta-analysis of randomized clinical trials. J. Prosthet. Dent..

[45] Kim D.H., Kim H.J., Kim S., Koo K.T., Kim T.I., Seol Y.J., Lee Y.-M., Ku Y., Rhyu I.-C. (2018). Comparison of marginal bone loss between internal- and external-connection dental implants in posterior areas without periodontal or peri-implant disease. J. Periodontal Implant. Sci..

[46] Meloni S.M., Lolli F.M., de Riu N. (2014). Platform switching vs. regular platform implants: Nine-month post-loading results from a randomised controlled trial. Eur. J. Oral Implantol..

[47] Enkling N., Jöhren P., Klimberg V., Bayer S., Mericske-Stern R., Jepsen S. (2011). Effect of platform switching on peri-implant bone levels: A randomized clinical trial. Clin. Oral Implant. Res..

[48] Hermann J.S., Schoolfield J.D., Schenk R.K., Buser D., Cochran D.L. (2001). Influence of the size of the microgap on crestal bone changes around titanium implants. A histometric evaluation of unloaded non-submerged implants in the canine mandible. J. Periodontol..

[49] Gardner D.M. (2005). Platform switching as a means to achieving implant esthetics. N. Y. State Dent. J..

[50] Lazzara R.J., Porter S.S. (2006). Platform switching: A new concept in implant dentistry for controlling postrestorative crestal bone levels. Int. J. Periodontics Restor. Dent..

[51] Baumgarten H., Cocchetto R., Testori T., Meltzer A., Porter S. (2005). A new implant design for crestal bone preservation: Initial observations and case report. Pract. Proced. Aesthet. Dent..

[52] Hahn J.A. (2007). Clinical and radiographic evaluation of one-piece implants used for immediate function. J. Oral Implantol..

[53] Tarnow D.P., Magner A.W., Fletcher P. (1992). The effect of the distance from the contact point to the crest of the bone on the presence or absence of the interproximal dental papilla. J. Periodontol..

[54] Tarnow D.P., Elian N., Fletcher P., Froum S., Magner A., Cho S.-C., Salama M., Salama H., Garber D.A. (2003). Vertical distance from the crest of bone to the height of the interproximal papilla between adjacent implants. J. Periodontol..

[55] Butera A., Gallo S., Pascadopoli M., Luraghi G., Scribante A. (2021). Ozonized Water Administration in Peri-Implant Mucositis Sites: A Randomized Clinical Trial. Appl. Sci..

[56] Bonato R.S., Fernandes G.V.O., Calasans-Maia M.D., Mello A., Rossi A.M., Carreira A.C.O., Sogayar M.C., Granjeiro J.M. (2022). The Influence of rhBMP-7 Associated with Nanometric Hydroxyapatite Coatings Titanium Implant on the Osseointegration: A Pre-Clinical Study. Polymers.

[57] Bratu E.A., Tandlich M., Shapira L. (2009). A rough surface implant neck with microthreads reduces the amount of marginal bone loss: A prospective clinical study. Clin. Oral Implant. Res..

[58] Piao C.M., Lee J.E., Koak J.Y., Kim S.K., Rhyu I.C., Han C.H., Herr Y., Heo S.J. (2009). Marginal bone loss around three different implant systems: Radiographic evaluation after 1 year. J. Oral Rehabil..

[59] Puchades-Roman L., Palmer R.M., Palmer P.J., Howe L.C., Ide M., Wilson R.F. (2000). A clinical, radiographic, and microbiologic comparison of astra tech and Branemark single tooth implants. Clin. Implant. Dent. Relat. Res..

[60] Al-Thobity A.M., Kutkut A., Almas K. (2017). Microthreaded Implants and Crestal Bone Loss: A Systematic Review. J. Oral Implantol..

[61] Palacios-Garzón N., Mauri-Obradors E., Roselló-LLabrés X., Estrugo-Devesa A., Jané-Salas E., López-López J. (2018). Comparison of Marginal Bone Loss Between Implants with Internal and External Connections: A Systematic Review. Int. J. Oral Maxillofac. Implant..

[62] Cha J.Y., Pereira M.D., Smith A.A., Houschyar K.S., Yin X., Mouraret S., Brunski J., Helms J. (2015). Multiscale analyses of the bone-implant interface. J. Dent. Res..

[63] Norton M. (2017). The Influence of Low Insertion Torque on Primary Stability, Implant Survival, and Maintenance of Marginal Bone Levels: A Closed-Cohort Prospective Study. Int. J. Oral Maxillofac. Implant..

[64] Esposito M., Maghaireh H., Pistilli R., Gabriella Grusovin M., Taek Lee S., Trullenque-Eriksson A., Gualini F. (2016). Dental implants with internal versus external connections: 5-year post-loading results from a pragmatic multicenter randomised controlled trial. Eur. J. Oral Implantol..

[65] Augusto G., Barbosa S., Almeida De Melo L., Bezerra De Farias D., Bezerra De Medeiros A.K., Dantas M., Carreiro A. (2017). Comparative evaluation of peri-implant tissues in patients wearing mandibular overdenture with different implant platforms. J. Indian. Soc. Periodontol..

[66] Castro F.M.C., Martins G.Z., Oliveira H.F.P., Hernández P.B., Gavinha S., Fernandes G.V.O. (2022). Comparison of Stud- Retentor Versus Bar-Clip Attachment as Implant- Supported Systems Used in Overdentures: A Systematic Review and Meta-Analysis. Eur. J. Prosthodont. Restor. Dent..

[67] Desai S.R., Koulgikar K.D., Alqhtani N.R., Alqahtani A.R., Alqahtani A.S., Alenazi A., Heboyan A., Fernandes G.V.O., Mustafa M. (2023). Three-Dimensional FEA Analysis of the Stress Distribution on Titanium and Graphene Frameworks Supported by 3 or 6-Implant Models. Biomimetics.

