# Supplementary material for: Marginal Bone Level and Clinical Parameter Analysis Comparing External Hexagon and Morse Taper Implants: A Systematic Review and Meta-Analysis
Source: Diagnostics (Basel). 2023 Apr 28;13(9):1587. doi: 10.3390/diagnostics13091587 (PMC10178059; doi:10.3390/diagnostics13091587)
Supplement: Supplementary file 1 [file diagnostics-13-01587-s001.zip › diagnostics-2280584-supplementary.pdf]

Table S1. Search strategy carried out and filters applied.

#1	P – patients rehabilitated with dental implants<br>(“Dental Implants" [MeSH Terms]) OR (Dental Implant* [Supplementary Concept])) OR (“Implant Placement”) OR (“Implant”)
#2	I – implant with external hexagon connection<br>(“External Hexagon”) OR (“External Connection”)
#3	C – implant with morse taper connection
	(“Internal Connection”) OR (“Morse Taper”) OR (“Morse”)
#4	O – clinical findings (MBL, BoP, inflammation, radiographic bone loss)
	(“Crestal Bone”) OR (“Marginal Bone”) OR (“Bone Loss”), (“Bleeding”) OR (“Bleeding on Probing”)
Search Combination	(#1 AND #2 AND #3 AND #4)
Filters	English, Humans
